# MC4R and ENPP1 gene polymorphisms and their implication in maternal and neonatal risk for obesity

**DOI:** 10.1038/s41598-019-47402-2

**Published:** 2019-07-26

**Authors:** Claudiu Mărginean, Cristina Oana Mărginean, Mihaela Iancu, Lorena Elena Meliț, Florin Tripon, Claudia Bănescu

**Affiliations:** 1 Department of Obstetrics and Gynecology, University of Medicine, Pharmacy, Sciences and Technology, Târgu Mureș, Gheorghe Marinescu street no 38, Târgu Mureș, 540136 Romania; 2 Department of Pediatrics, University of Medicine, Pharmacy, Sciences and Technology Târgu-Mureș, Gheorghe Marinescu street no 38, Târgu Mureș, 540136 Romania; 3 Department of Medical Informatics and Biostatistics, University of Medicine and Pharmacy Cluj Napoca, Victor Babes street no 8, Cluj Napoca, Romania; 4 Genetics Laboratory, Center for Advanced Medical and Pharmaceutical Research, University of Medicine, Pharmacy, Sciences and Technology Târgu-Mures, Gheorghe Marinescu street no 38, Târgu Mureș, 540136 Romania

**Keywords:** Paediatric research, Predictive markers

## Abstract

The *aims* of this study were to establish the role of *MC4R*rs17782313 *and ENPP1*rs1044498 gene polymorphisms on pre-pregnancy BMI and the newborn’s status. We performed a cross-sectional study on 185 mothers and their offspring. The groups were divided into: control group- underweight or normal mothers with BMI_initial_ < 25 kg/m^2^ (n_1_ = 134) and study group-overweight/obese mothers with BMI_initial_ ≥ 25 kg/m^2^ (n_2_ = 51). All subjects underwent demographic, anthropometric, paraclinical, bioimpedance and genetic parameters. We found association between initial BMI and gestational weight gain (GWG), and a higher frequency of excessive GWG in overweight/obese women (p = 0.037). Higher values of anthropometric and bioimpedance parameters were observed in overweight/obese versus underweight/normal women. The *MC4R* rs17782313 and *ENPP1* rs1044498 variant genotypes had an increased risk of pre-pregnancy overweight (OR = 1.41; 95% CI:[0.72; 2.78]; OR = 1.34; 95% CI:[0.65; 2.75]). The newborns from mothers with excessive GWG had a higher birth weight (BW) (p = 0.001). Higher MUAC values were noticed in newborns with *MC4R* rs17782313 wild-type genotype. Also, BW was correlated with GWG status smoking in pregnancy, gestational age and neonatal *ENPP1*rs1044498 variant genotype (p = 0.026). Our study pointed out the role of *MC4R* rs17782313 and *ENPP1* rs1044498 genotypes in obesity determinisms in mothers and their newborns in correlation with BMI, MUAC, TST and bioimpedance parameters.

## Introduction

Gestational weight gain (GWG) is an essential parameter of birth-related outcomes. Thus, the Institute of Medicine (IOM)^1^ defined the following recommendations regarding GWG: for underweight women (Body Mass Index - BMI < 18.5 kg/m^2^), recommended GWG 12.5–18 kg; normal weight women (BMI = 18.5–24.9 kg/m^2^), recommended GWG 11.5–16 kg; overweight women (BMI = 25–29.9 kg/m^2^), recommended GWG 7.00–11.5 kg; obese women BMI > 30 kg/m^2^, recommended GWG 5–9 kg15. Excessive gestational weight gain (GWG) in pregnant women is associated with diabetes, obesity, and dystocia^2,3^, but also modifications of neonatal birth weight (BW) with afterwards consequences on their evolution, such as macrosomia and obesity, and a wide range of other complications, like cardiovascular diseases, muscular and skeletal impairment, type 2 diabetes mellitus, and even psychological consequences^4^. World Health Organization data stated that according to the European Childhood Obesity Surveillance Initiative study, the incidence of increased weight among children from Romania is 26.8%, whereas that of obesity is 11.6%, being almost the highest incidence from all countries included in the study^5^. BW is another important parameter with impact on the newborn’s wellbeing^4^.

There are multiple genes involved in the determinism of obesity like: Leptin receptor *(LEPR)*^6^, Fat mass and obesity-associated gene *(FTO)*^7,8^, Single-minded homolog *(SIM1)* and Propiomelanocortin *(POMC)*^9^, Src homology 2B (*SH2B)* Adaptor Protein 1 gene, Peroxisome proliferator-activated receptor gamma gene *(PPAR-γ)*^10,11^, *IL-6* 572 (C > G, 190 C > T, and 174 G > C gene polymorphisms)^12,13^, angiotensin converting enzyme (*ACE I/D*), but also Tumor necrosis factor (*TNF) alfa* 308 G > A^14^.

Recently, other genes that are thought to have an impact in the development of obesity were discovered, such as: melanocortin receptor (*MCR*) *MC4R*, *MC3R*, *MC2R*, and also nucleotide pyrophosphatase/phosphodiesterase (*ENPP1*). The variant rs17782313 of *MC4R* is associated with obesity in both children and adults, regulating the control of energetic balance^15^. The *ENPP1* gene inhibits the sensitivity of insulin receptor, being considered a gene with a potential role in determining insulin-resistance and type 2 diabetes mellitus^16^.

Therefore, the aims of this study were to establish (i) the association between both *MC4R* rs17782313 and *ENPP1* rs1044498 gene polymorphisms and pre-pregnancy BMI and (ii) the impact of these 2 gene polymorphisms on excessive, respectively insufficient gestational weight gain, but also (iii) to establish if the *MC4R* rs17782313 and *ENPP1* rs1044498 single nucleotide polymorphisms (SNPs) were multivariate predictors correlated with birth weight, neonatal mid-upper-arm circumference and tricipital skinfold thickness in newborns.

## Results

### The demographic description of the studied groups regarding anthropometric, clinical, paraclinical and bioimpedance characteristics

Of all 225 mother-newborn couples that were examined, after applying the exclusion criteria, only 185 remained in the present study.

The mean age of mothers included in the study was 28.10 ± 5.80 years, over 50% of them having superior level education, 22.2% with education level <8 years, 17.8% with educational level between 9 and 12 years, and only 5.9% had never frequented school.

Most of the mothers included in our study were primipara (56.2%), with a GWG mean value of 17.1 ± 6.2 kg. Of all 185 pregnant women 11.4% were smokers, 5.90% presented preeclampsia and only 2.7% were diagnosed with gestational diabetes (Table 1).

**Table 1:** Comparison of maternal and neonatal characteristics and initial BMI.

	Study sample n = 185 (%)	Underweight/normal (Initial BMI < 25 kg/m^2^) n_1_ = 134 (%)	Overweight/obesity (Initial BMI ≥ 25 kg/m^2^) n_2_ = 51 (%)	p-value
**Maternal characteristics**				
Maternal age (years)	28.1 ± 5.8	27.5 ± 5.7	29.7 ± 5.8	0.017*
**Education**				
0 years	11 (5.9)	7 (5.2)	4 (7.8)	0.338
≤8 years	41 (22.2)	33 (24.6)	8 (15.7)	
9–12 years	33 (17.8)	26 (19.4)	7 (13.7)	
>12 years	100 (54.1)	68 (50.7)	32 (2.7)	
**Parity**				
0	104 (56.2)	76 (56.7)	28 (54.9)	0.824
≥1	81 (43.8)	58 (43.3)	23 (45.1)	
GWG (kg)	17.1 ± 6.2	17.5 ± 6.2	16.0 ± 6.0	0.137
**Status of GWG [n (%)]**				
Insufficient	27 (14.6)	25 (18.7)	2 (3.9)	0.037*
Normal	42 (22.7)	30 (22.4)	12(23.5)	
Excessive	116 (62.7)	79 (59.0)	37 (72.5)	
Smoking				0.067
No	164 (88.6)	115 (85.8)	49 (96.1)	
Yes	21 (11.4)	19 (14.2)	2 (3.9)	
**Hypertension in pregnancy**				
No	174 (94.1)	127 (94.8)	47 (92.2)	0.499
Yes	11 (5.9)	11 (5.2)	11 (7.8)	
Diabetes				0.021*
No	180 (97.3)	133 (99.3)	47 (92.2)	
Yes	5 (2.7)	1 (0.7)	4 (7.8)	
**Maternal bioimpedance parameters**				
MUAC (cm)	29.0[27.0; 32.0]	28.0[26.0; 30.0]	30.5[33.0; 35.5]	<0.001*
TST (mm)	18.0[13.0; 23.0]	15.6[11.0; 21.0]	24.0[18.5; 29.0]	<0.001*
FM (kg)	22.0[18.0; 28.0]	20.0[16.0; 23.1]	32.0[26.0; 38.0]	<0.001*
MM (kg)	46.0[42.0; 50.9]	44.0[41.0; 48.0]	51.0[48.0; 54.9]	<0.001*
BM (kg)	2.0[2.0; 3.0]	2.0[2.0; 2.7]	3.0[2.9; 3.0]	<0.001*
TBW (kg)	34.0[32.0; 38.0]	33.0[30.5; 36.0]	39.0[36.0; 41.8]	<0.001*
BMR (kcal)	1476.0[1387.0; 1644.0]	1430.0[1363.0; 1541.0]	1658.0[1568.5; 1776.5]	<0.001*
**Paraclinical parameters**				
ALAT (u/l)	11.0[8.0; 16.0]	11.5[8.0; 16.0]	11.0[8.0; 16.0]	0.796
ASAT (u/l)	22.0[18.0; 30.0]	23.0[19.0; 31.0]	22.0[17.0; 27.0]	0.333
Chol total(mg/dl)	212.0[194.0; 240.0]	211.0[194.0; 239.0]	222.0[196.0; 244.0]	0.381
HDL-chol (mg/dl)	59.0[50.0; 69.0]	59.0[51.0; 69.0]	60.0[48.0; 69.5]	0.920
LDL-chol (mg/dl)	134[113.0; 159.0]	134.0[113.0; 162.0]	135.0[114.5; 153.0]	0.942
TG (mg/dl)	200[169.0; 239.0]	191.5[158.0; 237.0]	223.0[187.0; 257.0]	0.049*
**Newborn characteristics**				
BW (kg)	3.4 ± 0.5	3.3 ± 0.5	3.5 ± 0.5	0.094
Apgar Score_1min	10[2; 10]	10[2; 10]	10[3; 10]	0.048*
Height (cm)	53.8 ± 2.6	53.8 ± 2.6	53.8 ± 2.5	0.939
BMI(kg/m^2^)	11.7 ± 1.1	11.5 ± 1.0	12.0 ± 1.2	0.003*
MUAC (cm)	11.0[10.0; 12.0]	10.3[10.0; 11.0]	11.0[10.0; 12.0]	0.059
TST (mm)	3.0[2.0; 3.0]	3.0[2.0; 3.0]	3.0[2.2; 3.0]	0.988
ALAT(u/l)	9.4[7.4; 12.0]	9.5[7.4; 11.8]	8.9[7.3; 12.4]	0.773
ASAT(u/l)	29.1[23.9; 36.4]	29.3[23.7; 36.9]	28.4[25.2; 34.9]	0.713
Chol (mg/dl)	82.1[58.9; 166.0]	97.4[61.5; 166.6]	72.7[53.9; 159.8]	0.148
HDL- chol (mg/dl)	38.1[25.3; 52.2]	39.5[27.3; 53.2]	32.0[24.5; 47.6]	0.118
LDL - chol (mg/dl)	41.1[24.0; 99.9]	49.2[25.6; 99.7]	32.2[19.4; 97.7]	0.100
TG (mg/dl)	51.6[30.7; 129.6]	52.5[30.7; 132.5]	47.1[31.5; 122.5]	0.849

Legend: ALAT: alanine aminotransferase, ASAT: aspartate aminotransferase, BM: Bone mass, BMI: Body mass index, BMR: Basal metabolism rate, BW: birth weight, Chol: cholesterol, FM: Fat mass, GWG: gestational weight gain, *HDL-chol: high density lipoprotein – cholesterol; LDL-chol: low density lipoprotein-cholesterol; MM: Muscle mass*, MUAC: Middle upper arm circumference, n - absolute number, SD - standard deviation, *TBW: Total body water*, *TG:* triglycerides, TST: Tricipital skinfold thickness.

Data expressed as mean ± standard deviation [percentile 25%; percentile 75%]; descriptive statistics for Apgar score were presented as median [minimum; maximum]; p-values obtained from Student-t test for independent samples or Mann-Whitney test or Chi-square test; *statistical significance (p < 0.05).

The comparison between maternal and neonatal characteristics reported to the body mass index at the beginning of the pregnancy is presented in Table 1. Thus, we found a significant association between initial BMI and maternal age (MAge) (p < 0.05), the overweight/obese mothers having a median age higher than those included in the control group.

We encountered a significant association between initial BMI and GWG, the frequency of excessive GWG being higher in overweight or obese women (72.5% versus 59%; p = 0.037) as compared to control group. Moreover, the incidence of diabetes mellitus was higher in obese mothers (7.8% versus 0.7%; p = 0.021).

Regarding anthropometric and bioelectrical impedance analysis (BIA) parameters, we found a significant difference between overweight/obese and underweight/normal weight women, identifying higher values of these parameters in overweight/obese women (Table 1).

Among the newborns’ characteristics, only BMI was significantly different in the newborns of overweight/obese mothers versus underweight/normal weight mothers (12.0 ± 1.0 versus 11.5 ± 1.2, p = 0.003).

### Gestational weight gain status and maternal gene polymorphisms

We found no statistically significant associations between *MC4R* rs17782313 variant genotype and increased weight/obesity before pregnancy (Table 2). After adjusting for maternal covariates as MAge, education level and parity, the presence of *MC4R* rs17782313 variant genotype (CT + CC) was associated with an increased risk of pre-pregnancy increased weight (adjusted OR = 1.41; 95% CI:[0.72; 2.78]). The same results were obtained for *ENPP1* rs1044498 (adjusted OR = 1.34; 95% CI:[0.65; 2.75]).

**Table 2:** Associations between MC4R rs17782313 and ENPP1 rs1044498 SNPs and GWG status.

	Study sample n(%)	Underweight/normal n(%)	Overweight/obesity n(%)	p^+^	Insufficient GWG n(%)	Normal GWG n(%)	Excessive GWG n (%)	p^+^
***MC4R*** **rs17782313 Maternal SNP**								
TT	106 (57.3)	81 (60.4)	25 (49.0)	0.185	17 (63.0)	25 (59.5)	64 (55.2)	0.721
CT + CC	79 (42.7)	53 (39.6)	26 (51.0)		10 (37.0)	17 (40.5)	52 (44.8)	
T-allele	285 (77.0)	212 (79.1)	73 (71.6)	0.124	41 (75.9)	67 (79.8)	177 (76.3)	0.793
C-allele	85 (23.0)	56 (20.9)	29 (28.4)		13 (24.1)	17 (20.2)	55 (23.7)	
p-value for HW^(b^	0.118	0.136	0.439		0.131	0.100	0.071	
***ENPP1*** **rs1044498 Maternal SNP**								
AA	129 (69.7)	95 (70.9)	34 (66.7)	0.576	19 (70.4)	28 (66.7)	82 (70.7)	0.886
AC + CC	56 (30.3)	39 (29.1)	17 (33.3)		8 (29.6)	14 (33.3)	34 (29.3)	
A-allele	310 (83.8)	226 (84.3)	84 (82.4)	0.645	46(85.2)	68 (80.9)	196 (84.5)	0.166
C-allele	60 (16.2)	42 (15.7)	18 (17.6)		16 (29.6)	16 (19.1)	36 (15.5)	
p-value for HW	0.640	0.849	0.571		0.366	0.634	0.574	

Legend: GWG = Gestational weight gain; ^(b^Hardy-Weinberg equilibrium; ^+^p-values obtained from Chi-square or Fisher’s exact tests.

ENPP1rs1044498 A > C: nucleotide pyrophosphatase/phosphodiesterase gene polymorphisms (AA = reference category); AA – homozygous for A allele; AC – heterozygous; CC - homozygous for C allele;

MC4R rs17782313 T > C: melanocortin- 4 receptor gene polymorphisms (TT = reference category); CC - homozygous for C allele; CT - heterozygous; TT- homozygous for T allele.

We found no significant associations between the variant genotype of *MC4R* rs17782313 and GWG status (Table 2). After controlling for maternal covariates such as MAge, educational level, parity, smoking during pregnancy, the presence of *MC4R* rs17782313 variant genotype (CT + CC) was not significantly associated with an increased risk of excessive GWG (adjusted OR = 1.16; 95% CI: [0.54; 2.48]) or insufficient GWG (adjusted OR = 0.96; 95% CI:[0.30; 3.06]). The same results were obtained for *ENPP1* rs1044498 SNP (excessive GWG: adjusted OR = 0.77; 95% CI: [0.34; 1.71] and insufficient GWG: adjusted OR = 0.89, 95% CI: [0.28; 2.84]).

The studied genotype frequencies of the two gene polymorphisms were consistent with Hardy-Weinberg equilibrium in the studied sample and GWG status (p > 0.05).

### Neonatal gene polymorphisms correlated with birth weight, mid-upper-arm circumference and tricipital skinfold thickness

In the studied sample (n = 185 newborns), the mean BW was 3368 g ± 448, with 9.7% (18 cases) above 4000 grams. We found no significant difference between the means of BW in newborns whose mothers had an initial BMI ≥25 kg/m^2^ (overweight/obese mothers) in comparison to those from mothers with an initial BMI <25 kg/m^2^. BW, middle upper arm circumference (MUAC) and tricipital skinfold thickness (TST) were significantly associated with maternal GWG status (one-way ANOVA, F(2; 182) = 8.19, p < 0.001). In the post-test analysis, the mean BW was significantly different in newborns whose mothers had insufficient or normal GWG in comparison to those from mothers with excessive GWG (Tukey test, p = 0.001). Therefore, the mothers with excessive GWG had newborns with higher BW (Table 3).

**Table 3:** Relationship between studied gene polymorphisms and neonatal anthropometric bioimpedance characteristics.

Factors	Birth weight (kg)		MUAC (cm)		TST (cm)	
	Mean ± SD	p^+^	Median[Q1; Q3]	p^+^	Median[Q1; Q3]	p^+^
**BMI initial (kg/m** ^2^ **)**						
<25	3.33 ± 0.45	0.094	10.5[10.0; 11.3]	0.075	2.8[2.3; 3.4]	0.912
≥25	3.46 ± 0.45		11.0[10.5; 11.5]		2.9[2.4; 3.2]	
**GWG status**						
Insufficient	3.11 ± 0.49	<0.001	10.3[9.7; 10.8]	0.005	2.3[2.0; 2.8]	<0.001
Normal	3.28 ± 0.43		10.5[10.2; 11.4]		2.8[2.4; 3.5]	
Excessive	3.46 ± 0.42		11.0[10.5; 11.5]		2.3[2.0; 2.8]	
***MC4R*** **rs17782313 Maternal SNP**						
TT	3.37 ± 0.43	0.892	11.0[10.3; 11.5]	0.149	2.9[2.3; 3.4]	0.519
CT + CC	3.36 ± 0.48		10.5[10.0; 11.5]		2.8[2.4; 3.5]	
***ENPP1*** **rs1044498 Maternal SNP**						
AA	3.35 ± 0.43	0.333	10.5[10.0; 11.3]	0.085	2.8[2.3; 3.2]	0.208
AC + CC	3.42 ± 0.49		11.0[10.3; 11.5]		3.0 [2.4; 3.4]	
***MC4R*** **rs17782313 Neonates SNP**						
TT	3.40 ± 0.45	0.250	11.0[10.3; 11.5]	0.035	2.8[2.3; 3.2]	0.346
CT + CC	3.32 ± 0.48		10.5[10.0; 11.2]		3.0 [2.4; 3.4]	
***ENPP1*** **rs1044498 Neonates SNP**						
AA	3.35 ± 0.42	0.538	10.5[10.0; 11.4]	0.105	2.8[2.3; 3.2]	0.123
AC + CC	3.40 ± 0.45		11.0[10.3; 11.5]		3.0 [2.3; 3.5]	

Legend: SD = standard deviation; Q1 = first quartile; Q3 = third quartile; ^+^p-values obtained from Student-t test for independent samples, ANOVA test or nonparametric test (as Mann-Whitney or Kruskal-Wallis).

BMI: Body mass index, BW: birth weight, MUAC: Middle upper arm circumference, SD: standard deviation, TST: Tricipital skinfold thickness.

ENPP1 rs1044498 A > C**:** nucleotide pyrophosphatase/phosphodiesterase gene polymorphisms (AA = reference category); AA – homozygous for A allele; AC – heterozygous; CC - homozygous for C allele;

MC4R rs17782313 T > C: melanocortin- 4 receptor gene polymorphisms (TT = reference category); CC - homozygous for C allele; CT - heterozygous; TT- homozygous for T allele.

Our study showed a significant difference regarding the distribution of MUAC values in newborns with *MC4R* rs17782313 variant genotype in comparison to those with normal genotype, higher values being noticed in the newborns with normal genotype (Table 3).

**Figure 1:**
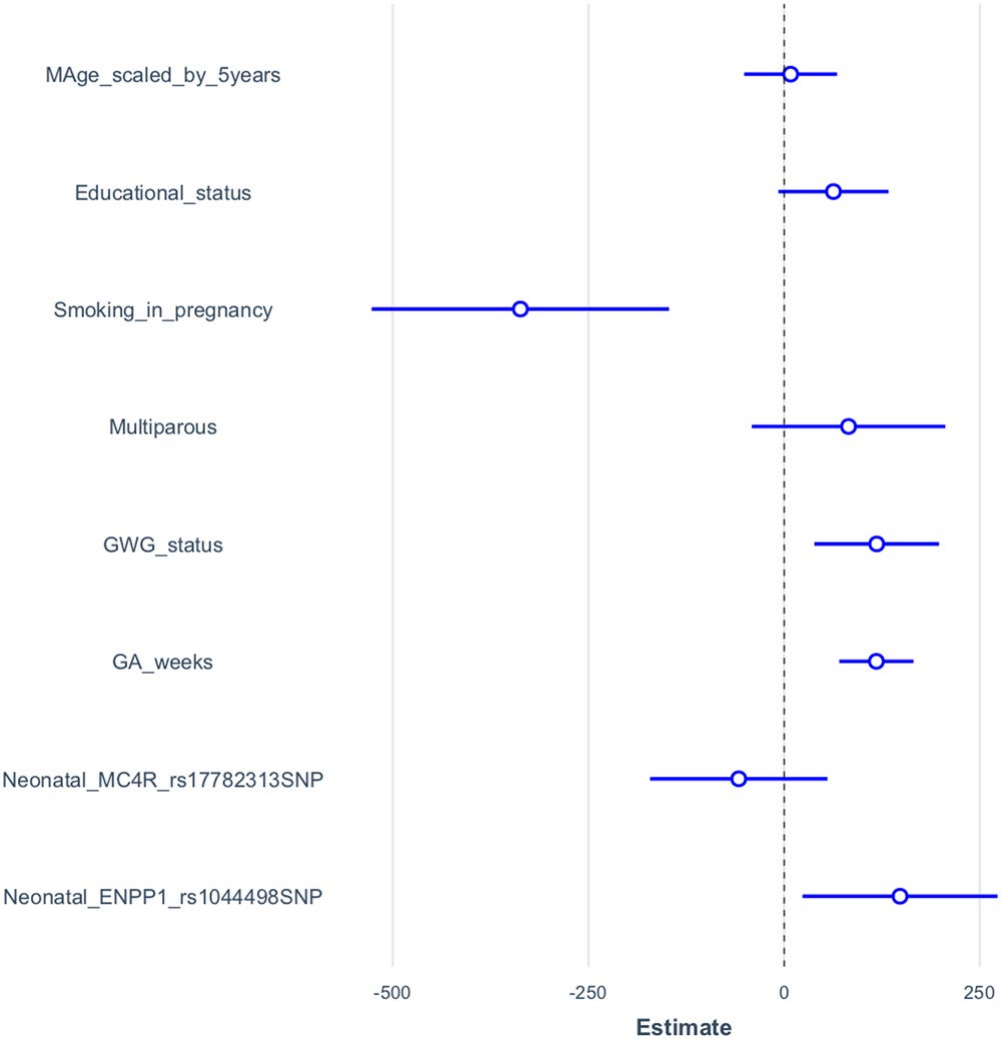
The effects of neonatal MC4Rrs17782313 and ENPP1rs1044498 SNPs on birth weight of full-term newborns adjusted for GWG status and other covariates - unstandardized regression beta coefficients with their confidence intervals (95% CI). Legend: Neonatal MC4Rrs17782313 and ENPP1rs1044498 SNPs were regarded as dichotomous predictors in multiple linear regression and coded as 1 = variant genotype, 0 = wild genotype; also for Multiparous (coded as 1 = yes, 0 = nulliparous) and Smoking in pregnancy (coded as 1 = yes; 0 = no) while Educational status (coded as 0 = 0 years of education; 1 = under 8 years of education; 2 = between 9 and 12 years of education; 3 = more than 12 years of education) and GWG status (0 = insufficient GWG; 1 = normal GWG; 2 = excessive GWG) were regarded as ordinal variables.

As a result of a linear multiple regression analysis, BW was significantly positively correlated with GWG status (p = 0.007), gestational age (GA) (p < 0.001) and neonatal *ENPP1* rs1044498 variant genotype (p = 0.026), while negatively correlated with smoking during pregnancy (p < 0.001). BW was positively correlated with excessive GWG status. Thus, for each unit increment in GWG status, BW increased by 113 g. The presence of neonatal variant *ENPP1* SNP was correlated with an increase in BW by 150 g. Moreover, an increase in BW by 132.1 g was observed per gestational week. The effect of GWG status, neonatal *ENPP1* rs1044498 and *MC4R* rs17782313 variant genotypes, adjusted according to important covariates (as mother’s age, educational level, smoking during pregnancy and gestational age at delivery) was illustrated in Fig. 1. Neonatal MUAC was significantly correlated with GWG status (p = 0.015), smoking in pregnancy (p = 0.024) and gestational age (GA) (p = 0.001). There was a tendency towards statistical association between neonatal *ENPP1* rs1044498 variant genotype and MUAC (p = 0.092). Regarding neonatal TST there was a significant correlation with GWG status (p = 0.001), and neonatal *ENPP1* rs1044498 variant genotype and MUAC (p = 0.045), the variant genotype being correlated with an increment in TST.

## Discussions

### Predictors for maternal weight gain

The studies reported in literature proved that maternal weight at the beginning of the pregnancy and GWG are important parameters that might result in diabetes mellitus, obesity and poor birth outcomes, with different obstetrical complications^2,3^. In addition, Farah *et al*. proved the fact that an excessive GWG leads to an increased neonatal BW with a subsequent risk of faster weight gain and even childhood obesity, but also other complications^4^. Similarly, in our study, BW was higher in newborns from overweight/obese women (3.5 ± 0.5) compared to underweight/normal weight group (3.3 ± 0.5).

Excessive GWG increases the risk for hypertension^17^, hypertriglyceridemia, obesity, type 2 diabetes mellitus, insulin resistance^18^, and is associated with increased BMI, TST, and waist circumference. Similarly, in our study, we noticed that excessive GWG and gestational diabetes incidence were higher in overweight or obese women (p = 0.037/p = 0.021).

Gallagher *et al*. proved that mothers with increased weight or obesity presented higher GWG and their newborns had a higher BW, but also a greater fat-free mass (FFM) evaluated by BIA^19^. Farah *et al*.^4^ and Butte *et al*.^20^ noticed a direct relationship between FFM and fat mass (FM) for GA under 37 weeks, with accurate predictions on BW. In our study, all anthropometric (MUAC, TST) and BIA (FM, MM, bone mass - BM, total body water - TBW and also basal metabolism rate - BMR) measurements were significantly higher in overweight/obese women than in underweight/normal weight women (p < 0.0001), similar to another study of the same authors^21^. Even though other studies^22^ proved that BW was correlated with total body water and FFM, but not with FM, in our study we noticed that BW was correlated with both FFM and FM.

There are studies proving that BW is correlated with GA at delivery^4,21^, but our study failed in proving this correlation. Contradictory data regarding the correlation between BW and smoking were reported by different studies. Thus, Farah *et al*. found a direct relationship between BW and smoking, and parity^4^, respectively, while Mărginean *et al*. in a previous study^21^ pointed out a reverse relationship between these parameters. The present study also underlined the negative impact of smoking during pregnancy on the newborn’s BW. Even though certain studies^11,21^ established a correlation between arterial hypertension (AHT) and higher GWG, in our study we did not notice this fact, probably due to the small number of cases with AHT.

Among the multiple *MCR* genes that proved to be involved in the etiology of obesity, MC4R is associated with monogenic obesity, regardless of age^15^. Therefore, the *MC4R* rs17782313 gene polymorphisms was associated in certain studies with obesity in both adults and children^15,23^. Thus, Bordoni *et al*.^24^ observed that the C/C genotype of *MC4R* rs17782313 gene polymorphism was associated with higher BMI and obesity risk in young Italian population. Similarly, Loos *et al*. emphasized that the C allele of the same gene was associated with an increased risk for developing obesity^15^, while Garcia-Solis *et al*. underlined a positive correlation between *FTO* rs9939609 homozygotes and *MC4R* rs17782313 heterozygotes and increased risk for both obesity and high blood pressure values in Mexican school-aged children^25^. Lazopoulou *et al*.^26^ proved that *MC4R* rs17782313 C allele was associated with a higher BMI and BW, but also that three or more high-risk alleles of the combined *FTO* and *MC4R* genotypes result in a 4 folds increased risk for obesity in Greek children and teenagers. Wu *et al*. noticed on Chinese children an association between *MC4R* rs17782313 SNP and adiponectin^27^.

Also, Mejía-Benítez *et al*.^28^ found associations between obesity risk, BMI and several SNPs, among which we recall *MC4R* rs17782313 and *ENPP1* rs7754561. Contrariwise, Albuquerque *et al*.^29^, Martins *et al*.^30^, similar to our study, identified no significant associations between *MC4R* rs17782313 SNP and GWG, maternal body weight, or other characteristic parameters for obesity.

*ENPP1* gene polymorphisms are associated with obesity in children, inhibiting insulin receptors and resulting in a higher incidence of type 2 diabetes mellitus and a lower tolerance for glucose in carriers of *ENPP1* K121Q (rs1044498) and A/G_1044TGA haplotypes^31,32^. Contrariwise, Morandi *et al*. stated that the Q121 variant allele of *ENPP1* K121Q (rs1044498) SNP presents a protective role for obesity in a study performed on 453 Italian children^33^. Other authors also underlined the relationship between the Q allele of *ENPP1* K121Q (rs1044498) SNP and type 2 DM or obesity^31,34^. In contradiction to the afore-mentioned studies, we found no correlations between *ENPP1* rs1044498 variant genotype and GWG status. Our findings are similar to those of Lyon *et al*.^35^ who did not notice any correlation between this SNP and obesity or diabetes mellitus.

### Predictors for newborn’s birth weight

One of the most important predictors for neonatal BW was proved to be excessive GWG. Therefore, the study of Ferrari *et al*. proved the fact that newborns coming from mothers with excessive GWG presented a higher BW in comparison to those whose mothers presented normal GWG^36^. The same study also underlined that the chances for these newborns to be macrosomic were 50% higher. Similarly, in our study, BW, MUAC and TST were correlated with GWG (p < 0.001), meaning that the newborns that came from mothers with excessive GWG had a higher weight than those from mothers with insufficient or normal GWG (p = 0.001). Some studies emphasized that newborns with higher BW are more predisposed to develop obesity and increased weight, but also more prone to cardiovascular and metabolic complications^37,38^. Previous studies performed by Farah *et al*.^4^ and Marginean *et al*.^21^ pointed out no correlation between initial BMI of the pregnant women and BW, similarly to the present study in which despite the fact that BW was with 9.7% higher in newborns from mothers with initial BMI ≥25 kg/m^2^ versus normal BMI, we found no significant difference between the two groups.

The studies of Bordoni *et al*.^24^ and Loos *et al*.^15^ pointed out the association between the variant allele C of *MC4R* rs17782313 and both increased BMI and obesity risk in teenagers. Also, the study of Lazopoulou *et al*.^26^ proved that BW and BMI were associated with the C allele of *MC4R* rs17782313, while Garcia-Solis *et al*.^25^ established a significant correlation between heterozygous *MC4R* rs17782313 carriers and both blood pressure and obesity risk in Mexican school-aged children^25^. Moreover, similar findings were underlined by other studies performed on Chinese people revealing a correlation between the *MC4R* rs17782313 SNP and adiponectin in Chinese obese children^27^. On the contrary, in our study, we noticed an association of the wild-type *MC4R* rs17782313 gene polymorphism and higher MUAC values in newborns.

It is well-documented that *ENPP1* gene is associated with obesity in pediatric patients resulting in an increased risk for glucose intolerance and type 2 diabetes mellitus^31^. Similarly, recent studies^32,39^ proved that the variant allele of *ENPP1* rs104449 increases the risk for obesity and type 2 diabetes mellitus, but no study established the role of this SNP in the newborn’s BW and pregnant woman’s nutritional status. On the other hand, Morandi *et al*. noticed that variant *ENPP1* rs104449 owns a protective role for developing obesity^33^. In exchange, our study proved a positive association between the same variant gene and GWG status, GA and an increase of 150 g in the mean value of the newborn’s BW. Moreover, a negative association was noticed regarding smoking during pregnancy and BW. Also, neonatal MUAC and TST were correlated with GWG and neonatal variant *ENPP1* rs1044498.

### Strengths and limitations of our study

The *strengths* of this study are the uniform population taken into consideration, the assessment of increased weight risk in mother-newborn couples, evaluating obesity risk in newborns and their correlations with other BIA and anthropometric parameters, and also evaluating the role of these genes in the determinism of BW. Some of the *limitations* of our study consist in the small number of mothers and newborns, the lack of assessment of other cytokines involved in the determinism of obesity, the type of diet, or the lack of BIA measurements in newborns.

There are a few data in literature that are meant to establish the role of these polymorphisms in the determinism of BW, therefore, we consider this study as a pilot one concerning the determinism of *MC4R* rs17782313 *and ENPP1* rs1044498 variant genotypes on GWG status and also on the mother-newborn couple.

## Conclusions

In our study we found a significant association between initial BMI and GWG, the frequency of excessive GWG being higher in overweight or obese women. The *MC4R* rs17782313 and *ENPP1* rs1044498 variant genotypes presented an increased risk in pre-pregnancy increased weight. Also, BW, MUAC and TST were significantly associated with GWG status. Higher MUAC values were noticed in newborns with wild-type *MC4R* rs17782313. The results of multivariate analysis showed that BW was positively correlated with GWG status, GA and neonatal variant *ENPP1* rs1044498, while negatively correlated with smoking during pregnancy.

Our study pointed out the role of *MC4R* rs17782313 and *ENPP1* rs1044498 in maternal and neonatal obesity risk in correlation with BMI, MUAC and TST and BIA, which could be useful for diagnosing obesity in mother-newborn couples.

## Methods

### Ethics approval and informed consent

The approval for this research was granted by the Ethics Committee of the University of Medicine, Pharmacy, Sciences and Technology from Târgu Mureș (No 26/7^th^ of April 2017), and it was performed according to the principles of the Helsinki Declaration. Informed consent was obtained from all individual participants included in the study (the mothers signed informed consent for them and their newborns).

#### Study sample selection

We developed a cross-sectional study on 185 mothers and their offspring, admitted in a Tertiary Hospital from Romania, in an Obstetrics Gynecology Clinic between May 2017 and October 2017. The groups were divided into: control group - the underweight or normal mothers with Initial BMI <25 kg/m^2^ (n_1_ = 134) and study group – the overweight/obese mothers with initial BMI ≥25 kg/m^2^ (n_2_ = 51).

The inclusion criteria were: MAge above 18 years and single pregnancies. The exclusion criteria were: intrauterine growth retardation as a result of congenital malformations, chronic disorders, intrauterine infections; the lack of complete clinical, paraclinical, anthropometric and genetic data, but also mothers who refused to sign the informed consent prior to the inclusion in the study.

#### Variables of interest

Body mass index (BMI), Middle upper arm circumference (MUAC), Tricipital skinfold thickness (TST), Fat mass (FM): We performed all measurements in both mothers and newborns. These measurements included: weight (kg), height (cm), MUAC and TST. Height was measured with a daily calibrated pedometer, being evaluated by SD (0.1-cm error). For MUAC determination, we measured the arm circumference with a tape measure calibrated in centimeters, at the midpoint between shoulder and elbow tips, while TST was assessed in the posterior area of the upper arm with a thickness caliper. BMI was calculated as the ratio between weight (kg) and squared standing height (m^2^). According to Control Disease Center (CDC), a BMI between 25.0 and 29.9 classified mothers as overweight, while those with a BMI of 30.0 or higher were considered obese.

For the bioelectrical impedance analysis (BIA), we used a Tanita BC-420 MA body composition analyzer (Tanita Corp, Tokyo, Japan). Weight was provided automatically with a 0.5 kg adjustment for the clothes weight, while height, sex, and age were typed manually.

#### Genetic variables

The DNA was isolated from fresh peripheral blood using the PureLink Genomic DNA kit (ThermoScientific) according to the manufacturer instructions. The concentration and the purity (*A*_260_/*A*_280_) of the DNA was quantified by spectrophotometry (BioSpectometer basic, Eppendorf).

For genotyping of *MC4R* rs17782313 and *ENPP1* rs1044498 we used TaqMan technology. In this respect we used the following single tub TaqMan SNP Genotyping assay formats: C__32667060_10 for rs17782313, and C__1207994_20 for rs1044498 (from ThermoFisher Scientific). Genotyping was performed according to the standard protocol for genotyping for 7500 Fast Dx Real-Time PCR System from Applied Biosystems. For genotypes interpretation the 7500 Fast Software v2.3 (Applied Biosystems) was used. All gDNA samples isolated from whole blood collected from children included in patient and control groups were successfully genotyped.

#### Statistical analysis

Qualitative variables were described by relative frequency while description of quantitative variables were released using centrality measures as mean standard deviation or median (interquartile range).

Bivariate associations between the studied gene polymorphisms, maternal GWG and neonatal characteristics (BW, MUAC, TST) were tested by Chi-square test, Student-t test for independent samples, ANOVA test or nonparametric tests (as Mann-Whitney or Kruskal-Wallis).

The effect of the studied gene polymorphisms on GWG and neonates’ characteristics (BW, MUAC, TST) was adjusted for other covariates using logistic regression analysis or linear multiple regression.

Statistical significance was achieved with the estimated significance level of p < 0.05. All statistical analysis was performed using environment for statistical computing R version 3.4.4.

### What is known

- Excessive gestational weight gain (GWG) is associated with diabetes, obesity, and dystocia, but also modifications of neonatal birth weight (BW) with afterwards consequences on their evolution. One of four Romanian children is obese or overweight, with percentages varying between 19.7% and 35.8%, depending on the geographic area
- *MC4R* and *ENPP1* gene polymorphism are associated with increased risk for developing obesity.

### What is new

- The *MC4R* rs17782313 and *ENPP1* rs1044498 variant genotypes presented an increased risk in pre-pregnancy overweight.
- BW was positively correlated with GWG status, gestational age and neonatal variant *ENPP1* rs1044498, while negatively correlated with smoking during pregnancy.
